# Integrating clinical evidence with natural product therapies for elderly-onset type 2 diabetes 

**DOI:** 10.3389/fphar.2025.1658881

**Published:** 2025-11-25

**Authors:** Chae-Eun Kim, Kyong Kim, Eun-Young Park, Yoon Sin Oh

**Affiliations:** 1 Department of Food and Nutrition, Eulji University, Seongnam, Republic of Korea; 2 College of Pharmacy and Natural Medicine Research Institute, Mokpo National University, Jeonnam, Republic of Korea

**Keywords:** natural compounds, aging, type 2 diabetes, diabetic complications, hyperglycemia

## Abstract

Elderly-onset type 2 diabetes presents a distinct clinical profile, typically characterized by milder hyperglycemia and specific risks for complications. Age-related physiological changes and increased sensitivity to side effects often limit the use of conventional medications in this population. This review summarizes the epidemiology, clinical characteristics, and management challenges of diabetes in older adults and explores the therapeutic potential of natural bioactive compounds—including *Enteromorpha prolifera*, *Ficus* species, genipin, gingerol, mulberry, myricitrin, quercetin, resveratrol, and saffron. These findings not only provide mechanistic insights into the role of natural bioactive compounds in diabetes management but also highlight their clinical relevance, suggesting potential applications as adjunctive therapies for elderly individuals with limited pharmacological tolerance, and guiding future research toward evidence-based integration of such agents into clinical practice.

## Introduction

As global populations continue to age, the prevalence of type 2 diabetes mellitus (T2DM) among older adults has become a major public health concern. Adults aged 65 years and older represent the fastest-growing demographic of people living with diabetes worldwide, with estimates indicating that more than one in four individuals in this age group are affected (Kirkman et al., 2012). According to 2019 statistics from the American Diabetes Association, 37.3 million Americans—approximately 11.3% of the population—had diabetes. Among older adults aged 65 and older, the prevalence was notably higher at 29.2%, representing 15.9 million individuals. These numbers are globally projected to exceed 200 million by 2045 (American Diabetes Association, 2023). This increasing trend reflects both the cumulative metabolic burden over the lifespan and age-related physiological changes that predispose older individuals to impaired glucose regulation.

Elderly-onset T2DM shows a distinct clinical and pathophysiological profile compared to diabetes diagnosed earlier in life. In this population, postprandial hyperglycemia is typically more pronounced than fasting hyperglycemia, primarily due to impaired early-phase insulin secretion and a diminished incretin effect (Meneilly and Elahi, 2005). While insulin resistance remains a contributing factor, age-related declines in pancreatic β-cell function play a more central role in disease pathogenesis (Chang and Halter, 2003) (Table 1). Additional age-related factors—such as sarcopenia, increased visceral adiposity, chronic low-grade inflammation (“inflammaging”), and altered hormonal regulation—further contribute to metabolic dysregulation (Franc et al., 2014). Moreover, polypharmacy, cognitive decline, and comorbidities (e.g., cardiovascular disease, renal impairment) complicate diabetes management in older adults and may mask typical hyperglycemic syndromes, leading to delays in diagnosis and treatment (Abdelhafiz et al., 2015). These complexities highlight the urgent need for age-specific strategies in the prevention, diagnosis, and management of T2DM in this population. Understanding these features of elderly-onset diabetes is essential for developing personalized therapeutic approaches that optimize glycemic control while preserving quality of life and minimizing the risk of hyperglycemia-related complications and treatment-induced hypoglycemia (Table 2).

**TABLE 1:** A Comparison between of Young-onset diabetes and Elderly-onset diabetes.

	Young-onset	Elderly-onset
Glycemic profile	↑ High fasting and postprandial glucose	Increased postprandial glucose, relatively lower insulin secretion
Beta-cell function	Rapid decline	Slow decline
Insulin resistance	Marked (mainly obesity-related)	Mild (age-related factors)
Microvascular complication risk	↑ Higher from diagnosis	Lower or delayed
Macrovascular complication risk	↑ Higher in the long term	Higher early, more rapid progression

↑ denotes a relatively higher value or increased risk in comparison with the elderly-onset group.

**TABLE 2:** Personalized treatment strategy for Elderly Diabetes.

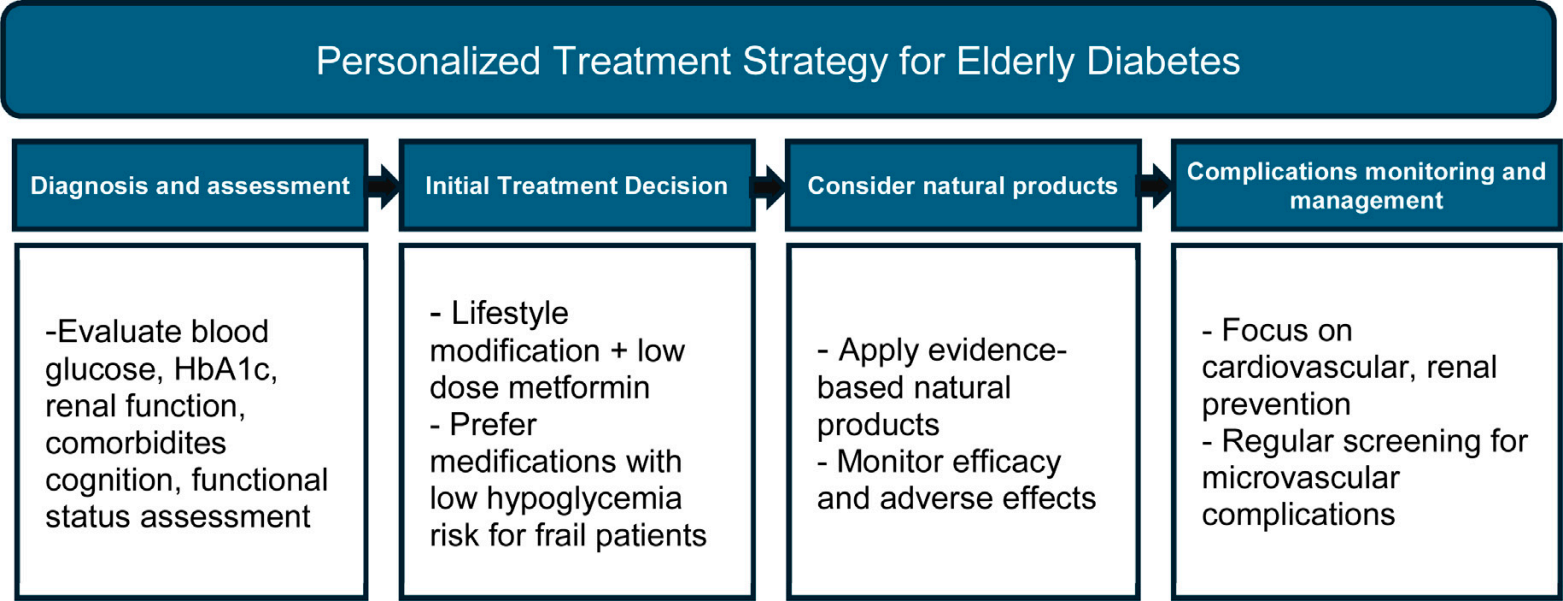

In this review, we aimed to provide a comprehensive and accessible synthesis on elderly-onset diabetes and natural product-based interventions. Given the limited availability of integrative reviews on this topic, we incorporated evidence from clinical, animal, and cellular studies to emphasize their potential applications in practice. For this, we summarize the characteristics of age-related diabetes and explores the potential of natural bioactive compounds for its prevention and treatment. We focus on the mechanisms of action and therapeutic effects of *Enteromorpha prolifera* oligosaccharides, *Ficus* species, genipin, gingerol, myricitrin, mulberry, quercetin, resveratrol, and saffron in age-related diabetes and its complications (Table 3; Figure 2).

**TABLE 3:** Summary diagram of the mechanisms of action of major bioactive natural products.

Bioactive compounds	Main target pathways	Glycemic control effect	Complication prevention effect	Reference
*Berberine*	Activate AMPK, suppress NF-κB, insulin receptor expression, mitochondrial dysfunction	Improve glucose metabolism Insulin sensitivity, β-cell protection reduce inflammation	Prevent nephropathy, retinopathy and neuropathy	10–19
*Enteromorpha prolifera*	Activate PI3K, JNK pathway Increase GLP-1 expression	Reduce oxidative stress Improve insulin resistance Reduce gut dysbiosis	Regulation of brain-gut axis	20–22
*Ficus* spp.	Increase antioxidant level Increase Ca^2+^ signaling Suppress a-amylase and DPP-4 activity	Promote beta-cell regeneration Improve lipid profile	Improve renal function Prevent neuropathy	23–36
*Genipin*	Modulate JNK and AKT signaling Suppress inflammatory cytokines Decrease ROS level, enhance ATP synthesis	Protect diabetic retinopathy Glucose control Improve implant osseointegration	Neuropathy prevention	37–43
*Gingerol*	Activate PPARα, CPT1α Increase mitochondrial marker enzyme Improve NO, suppress ROS	Reduce lipid synthesis Glucose regulation Protect diabetic complications	Prevent vascular and neural complications	44–54
*Mulberry*	Inhibit ROS production Scavenge free radical activity Increase AGE-RAGE signaling	Improve insulin resistance Reduce glucose levels Enhance beta cell viability	Prevent nephropathy, and retinopathy	55–71
*Myricitrin*	Decrease inflammatory cytokines Activate Nrf2/inhibit NF-kB pathway	Cardioprotective effect Anti-fibrotic effect	Prevent neuropathy and retinopathy	72–76
*Quercetin*	Decrease p16INK4A Suppress inflammatory cytokines/miR-155-5p	Anti-oxidant/Anti-inflammatory effect	Prevent nephropathy, vascular damage, and nerve function	77–91
*Resveratrol*	Activate AMPK Reduce inflammation markers Activate SIRT1/inhibit NF-kB	Improve insulin sensitivity Reduce oxidative stress Enhance mitochondrial function	Protection of nephropathy, neuropathy and retinopathy	92–101
*Saffron*	Increase adiponectin/decrease TNF-a Activate PI3K/AKT	Potential to modulate insulin and lipid metabolism Reduce oxidative stress protect diabetic retinopathy	Neuro- and retinopathy protective effect	102–110

## Literature searching

**FIGURE 1:**
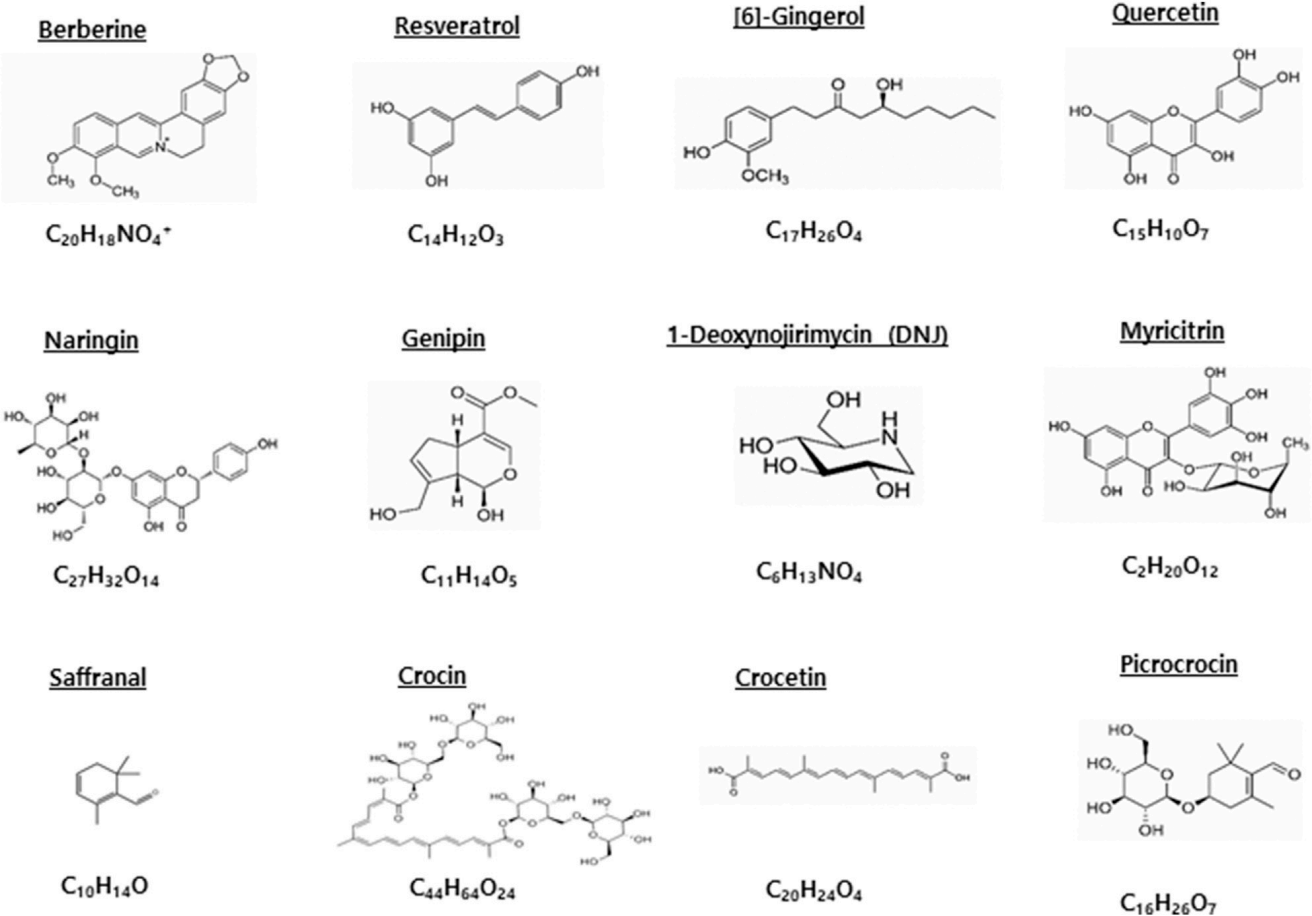
Chemical structures and molecular formulas of representative bioactive compounds with antidiabetic effects on elderly diabetes.

We first identified candidate compounds through searches using the keywords *“antidiabetic,” “natural compounds,”* and *“natural bioactives.”* From this pool, we prioritized those supported by convincing pharmacological evidence demonstrated in preclinical studies using aging-dependent diabetic animal models, as well as compounds that showed significant efficacy in clinical studies conducted among elderly populations. In addition, we considered only compounds with well-established antidiabetic mechanisms, such as inhibition of α-glucosidase or DPP-IV, activation of energy-sensing pathways like AMP-activated protein kinase (AMPK) and NAD-dependent deacetylase sirtuin-1 (SIRT 1), or protection of pancreatic β-cell function. Finally, we emphasized agents with substantial translational potential, grounded in evidence from traditional medicinal use or dietary safety. Collectively, the selected compounds represent structurally diverse groups—including marine polysaccharides, iridoid aglycones, phenolic flavonoids and glycosides, stilbenoids, and carotenoids—that are relevant to metabolic disease and particularly to diabetes associated with aging. The chemical structures of selected compounds are shown in Figure 1 (Figure 1).

In the literature search process, we primarily conducted traditional searches using the MEDLINE/PubMed database, applying specific keyword combinations and selection criteria. Additionally, to enhance the efficiency of information retrieval and literature summarization, we utilized AI-based tools such as Perplexity as a supplementary resource. The summaries and search results provided by the AI tool were carefully reviewed and incorporated into the final selection and analysis at the discretion of the researchers.

We excluded letters, abstracts, and conference proceedings that were not published in full in peer-reviewed journals. Titles and abstracts were screened to identify studies examining the association between bioactive food components and age-related diabetes.

## Natural products effective for age-related diabetes and its complications

### Berberine

Berberine, derived from species such as *Berberis vulgaris*, *Coptis chinensis*, and *Hydrastis canadensis*, exerts multiple metabolic benefits including glycemic control, lipid regulation, and inflammation reduction primarily via AMPK activation, microbiota modulation, and antioxidant mechanisms.

Although numerous studies have investigated the effects of berberine in patients with diabetes, research specifically involving elderly individuals remains limited. Yin et al. (2008a) demonstrated in a randomized controlled trial involving type 2 diabetic patients (age 25–75 years), oral administration of berberine (1.5 g/d for 3 months) significantly reduced fasting blood glucose, postprandial glucose, and HbA1c levels, with efficacy comparable to metformin (Yin et al., 2008a). Systemic review and meta-analysis study by Cao et al. (2019) reported that berberine treatment was associated with a better reduction of fasting blood glucose and HbA1c levels, but the effect may diminish in patients older than 60 years and with longer treatment duration, indicating age-dependent variability in response (Liang et al., 2019). Wang et al. (2024) also demonstrated that berberin effect was less pronounced in older adults and in longer-duration studies, suggesting age-specific metabolic responses (Wang et al., 2024).

Animal models of diabetes have demonstrated that berberine not only reduced hyperglycemia but also mitigates diabetes-induced cognitive impairment and oxidative stress. Meta-analyses of diabetes cognitive impairment models indicate berberine improves fasting blood glucose and memory function, likely through mechanisms involving insulin resistance improvement, anti-oxidant and anti-neuroinflammation (Hao et al., 2022). Additionally, in diabetic *db/db* mice, berberine ameliorates advanced glycation end product (AGEs)-induced ferroptosis (a form of regulated cell death linked to lipid peroxidation) in keratinocytes by activating the nuclear factor erythroid 2-related factor 2 (Nrf2) pathway, thus protecting skin tissue from diabetic damage (Jiang et al., 2024).

Berberine exerts significant antidiabetic effects in various cell models by AMPK, a key regulator of cellular energy metabolism. In adipocytes, muscle cells, and hepatocytes, berberine enhances glucose uptake and glycolysis, suppresses hepatic gluconeogenesis, and inhibits lipogenesis primarily through AMPK phosphorylation (Yin et al., 2008b; Li et al., 2015). Additionally, it increases glucose transporter-4 (GLUT4) expression and translocation, promoting insulin dependent glucose utilization (Cicero and Tartagni, 2012). Moreover, berberine demonstrates anti-oxidant and anti-inflammatory properties, reducing oxidative stress and inflammation implicated in insulin resistance and beta-cell dysfunction (Cicero and Tartagni, 2012). Recent studies also reveal its protective role against ferroptosis in pancreatic β-cells, suggesting preservation of insulin secretion capacity (Yu et al., 2025).

Berberine has demonstrated significant protective effects against various diabetic complications. In diabetic nephropathy, it improves renal function markers such as blood urea nitrogen, serum creatinine, and proteinuria through its anti-inflammatory, anti-fibrotic, and antioxidant actions, although further large-scale clinical trials are warranted (Hu et al., 2022; Pupyshev et al., 2022). In diabetic neuropathy, berberine, often combined with antioxidants like tocopherol, reduces oxidative stress and neuroinflammation by inhibiting NF-κB signaling, thereby alleviating nerve damage and neuropathic pain (Alkholifi et al., 2023). Additionally, berberine protects against diabetic retinopathy by suppressing pathological retinal neovascularization and endothelial cell activation via modulation of the Akt/mTOR/HIF-1α/VEGF pathway, helping to preserve retinal vascular integrity and prevent vision loss in experimental models (Wang et al., 2021).

These combined effects position berberine as a promising multi-targeted agent for type 2 diabetes management, warranting further investigation to optimize its clinical applications.

### *Enteromorpha prolifera* oligosaccharide (EPO)

Marine algae are a rich source of bioactive natural products. EPO, derived from green algae of the genus *Ulva*, is commonly found in the intertidal zones of oceans, particularly in regions such as China, the Baltic Sea, and Chile. The antidiabetic effects of EPO on age-related T2DM have been primarily studied in animal models, with no human clinical trials reported to date. In one study, diabetes was induced in aged mice using D-galactose (100–200 mg/kg) followed by streptozotocin (STZ, 45 mg/kg). Co-treatment with EPO (150 mg/kg) significantly improved glucose tolerance and enhanced superoxide dismutase (SOD) activity. EPO also modulated key metabolic pathways, including the tricarboxylic acid cycle and arginine- and inosine-related pathways in the brains of aged diabetic mice. These effects were linked to the upregulation of *daf-1* and *skin-1*, genes associated with lifespan extension in *Caenorhabditis elegans.*

Moreover, EPO increased the expression of GLP-1 in the gut, leading to enhanced GLP-1 receptor expression in the brain. This suggests that EPO may regulate glucose metabolism through the brain–gut axis (Ouyang et al., 2022). Zhu et al. also demonstrated that *E. prolifera* polysaccharide (EPP) improved glucose metabolism and suppressed aging-related gene expression in diabetic mice, likely through its probiotic effects and promotion of beneficial gut microbiota (Zhu et al., 2023).

In another study, a 55% ethanolic extract of *E. prolifera* showed hypoglycemic effects by activating phosphatidylinositol 3-kinase (PI3K) and suppressing c-Jun N-terminal kinase (JNK) signaling in the liver (Lin et al., 2018). These multifaceted mechanisms suggest that EPO may provide comprehensive preventive benefits for age-related diabetes by simultaneously targeting key factors such as insulin resistance, oxidative stress, and gut dysbiosis. However, clinical validation in humans remains essential before practical applications can be recommended.

### *Ficus* species

The genus *Ficus*, belonging to the Moraceae family, comprises over 800–900 species of woody trees, shrubs, vines, and hemiepiphytes (Deepa et al., 2018). These plants contain a diverse array of bioactive compounds with therapeutic potential against diabetes and its complications through multiple molecular pathways. Notably studied species include *Ficus benghalensis* L., *F. carica* L., *F. racemosa* L., *F. hispida* L., *F. microcarpa* L.F., *F. religiosa* L., *F. thonningii* Blume, *F. glumosa* Del., *F. arnottiana* Miq., *F. glomerata* Roxb., *F. sycomorus* L., and *F. deltoidea* Jack. Among these, *F. racemosa* and *F. carica* are the most extensively studied, with their extracts and isolated compounds showing validated antidiabetic activity (Stephen Irudayaraj et al., 2017; Liu et al., 2022).

*Ficus* species are rich in bioactive metabolites, including flavonoids, phenolic acids, tannins, alkaloids, glycosides, coumarins, triterpenoids, sterols, and vitamin E. These compounds have demonstrated hypoglycemic effects primarily by enhancing insulin secretion and improving glucose utilization in both *in vitro* and *in vivo* studies (Deepa et al., 2018).

In a small-scale study (n = 59), elderly patients (aged over 80 years) with diabetes after COVID-19 infection were administered *F. pumila* leaf extract (200 g/day) for 3 months. The treatment improved insulin secretion capacity (HOMA-β) and insulin resistance (HOMA-IR). However, the study’s small sample size and lack of long-term follow-up limit the generalizability of its findings (Gonda et al., 2025). Similarly, a double-blind clinical trial in T2DM patients (aged 40–60 years) found that daily consumption of *F. carica* leaf extract for 21 days significantly reduced fasting blood glucose, postprandial glucose, and HbA1c levels (Ahmadi Mazhin et al., 2016).

Li et al. reported that *F. vasculosa* ethanol extract (FVEE) showed protective effects against D-galactose-induced aging by reducing oxidative stress (Li et al., 2019a). Pretreatment with FVEE (200 mg/kg) increased serum and tissue antioxidant levels, including SOD, catalase, glutathione, and malondialdehyde. FVEE also showed higher reducing power and α,α-diphenyl-β-picrylhydrazyl radical-scavenging activity than vitamin C, with naringin identified as the most active antioxidant compound in the extract.

Sterols isolated from *F. racemosa* leaves (150 mg/kg) normalized blood glucose levels in alloxan-induced diabetic rats, restored lipid profiles (lowered low-density lipoprotein [LDL] and triglycerides, elevated high-density lipoprotein [HDL]), and reduced oxidative stress in both pancreatic and hepatic tissues (Sophia et al., 2007). Additionally, *F. carica* extract was shown to promote pancreatic β-cell regeneration in alloxan-induced diabetic rats (Saleem et al., 2023). The triterpene ficusonolide (50 mg/kg) significantly reduced blood glucose levels in STZ–nicotinamide-induced diabetic rats and inhibited key enzymes including α-glucosidase and DPP-4 (Din et al., 2021).

Methanolic extract from *F. deltoidea* enhanced insulin secretion in pancreatic β-cells via modulation of K^+^-ATP channels and intracellular Ca^2+^ signaling pathways (Akhtar et al., 2018). Meanwhile, acetone extract from *F. lutea* increased glucose uptake in primary muscle cells and hepatoma cells through activation of the glucose transporter (GLUT)-4 transporter (Adam et al., 2012). Additionally, *F. microcarpa* demonstrated multi-target enzyme inhibitory effects by suppressing α-amylase and DPP-4 activities (Akhtar et al., 2018).

Collectively, these findings suggest that *Ficus* species exert multimodal antidiabetic effects: human trials provide evidence of glucose-lowering efficacy for *F. pumila* and *F. carica*; animal studies reveal β-cell regeneration and lipid profile improvement for *F. racemosa* and *F. carica*; and cellular models clarify insulin-sensitizing and enzyme-inhibitory mechanisms for *F. lutea* and *F. microcarpa*. Further clinical validation is necessary to support broader therapeutic applications.

Ficus species, particularly *Ficus glomerata* and *Ficus carica*, have shown promising effects in alleviating diabetic complications. Studies have demonstrated that *Ficus glomerata* leaf extract improves renal function markers such as serum creatinine and blood urea nitrogen, while also reducing neuropathic symptoms like thermal hyperalgesia and cold allodynia in streptozotocin-induced diabetic animal models. These benefits are largely attributed to the plant’s antioxidant and anti-inflammatory properties (Shaikh et al., 2020). Additionally, *Ficus carica* seed oil, rich in anti-inflammatory and antioxidant compounds, has been reported to protect against diabetic neuropathy by regulating blood glucose levels and preserving nerve conduction and pain perception (Serap Oktay et al., 2024). Together, these findings highlight the neuroprotective and nephroprotective potential of Ficus species in managing diabetic complications.

### Genipin

Genipin is an aglycone derived from geniposide, an iridoid glucoside extracted from the fruit of *Gardenia jasminoides Ellis*, traditionally used in Asian medicine for its choleretic, hepatoprotective, and hypoglycemic effects (Tseng et al., 1995; Koo et al., 2004). Although several studies have reported genipin’s effects on aging-related diabetes and its complications, clinical trial data in humans remain limited.

In one study, intraperitoneal administration of genipin (25 mg/kg for 12 days) to 18-month-old rats significantly reduced hyperinsulinemia and hyperglycemia compared to controls. Genipin also alleviated palmitate-induced reactive oxygen species (ROS) overproduction and mitochondrial membrane potential loss in hepatocytes, potentially by modulating JNK and AKT signaling pathways (Guan et al., 2013).

In high-fat diet (HFD)-induced obese mice, genipin treatment (50 mg/kg for 6 weeks) improved lipid metabolism and reduced hepatic lipid accumulation (Wu et al., 2023). Furthermore, genipin demonstrated protective effects against diabetic retinopathy and vision loss induced by STZ, high sugar, and HFD feeding; treatment (10 mg/kg/day for 3 months) decreased vascular endothelial growth factor expression and suppressed inflammatory cytokines such as tumor necrosis factor (TNF)-α and interleukin (IL)-1β (Sun et al., 2023). In human retinal microvascular endothelial cells, 0.4 µM genipin reduced high glucose-induced apoptosis, decreased ROS levels, and enhanced adenosine triphosphate (ATP) synthesis (Sun et al., 2023).

Recently, combined treatment with genipin (50 mg/kg) and insulin (10 IU/kg) improved implant osseointegration and glycemic control in STZ-induced diabetic rats, effects that were associated with activation of AMPK signaling (Zhang et al., 2021a). Most research on genipin has been conducted using cell cultures or animal models, where its anti-inflammatory and antioxidant properties show promise in alleviating diabetes-related complications. However, geniposide—the precursor of genipin—has been reported to cause hepatotoxicity at high doses, and genipin itself has exhibited potential genotoxicity (Xia et al., 2021). Therefore, thorough safety evaluations are essential before genipin can be considered for therapeutic use.

In diabetic nephropathy, genipin has been shown to preserve renal function by reducing podocyte injury and albuminuria. This protective effect is largely achieved through the downregulation of mitochondrial uncoupling protein 2 (UCP2), which mitigates oxidative stress and inflammation in kidney cells. It also improves insulin sensitivity by stimulating glucagon-like peptide-1 (GLP-1) secretion from intestinal L-cells via the phospholipase C (PLC)/calcium signaling pathway (Wu et al., 2023).

Collectively, these multifaceted mechanisms highlight genipin’s potential as a promising therapeutic agent for managing type 2 diabetes and its associated complications.

### Gingerol

6-Gingerol is the major pharmacologically active component of ginger (*Zingiber officinale* Roscoe, Zingiberaceae) and exhibits a range of beneficial effects, including antioxidative, anti-inflammatory, and hepatoprotective properties (Nicoll and Henein, 2009; Abolaji et al., 2017). Clinical trials involving healthy adults consuming ginger powder (3 g/day) for 3 months demonstrated significant improvements in fasting blood glucose, HbA1c levels, and insulin resistance indicators such as HOMA-IR (Shidfar et al., 2015). Another study reported that intake of 1.2 g/day of ginger powder for 90 days reduced fasting blood glucose and total cholesterol levels in patients with T2DM (aged 20–80 years), indicating its potential as an adjunctive treatment (Carvalho et al., 2020).

In animal models, treatment with 6-gingerol (0.05–0.20 mg/kg) markedly normalized hepatic triglyceride accumulation, plasma insulin, and HOMA-IR in 22-month-old rats. The compound modulates lipid metabolism by activating peroxisome proliferator-activator receptor (PPAR)α and carnitine palmitoyltransferase (CPT)1α while inhibiting diacylglycerol O-acyltransferase (DGAT)-2, thereby enhancing β-oxidation and reducing lipid synthesis (Liu et al., 2019).

Furthermore, 6-gingerol reversed reductions in citrate synthase (Cs) and ATP, decreased ROS-induced damage, and upregulated mitochondrial marker enzymes including nitrogen oxides (NOX), succinate dehydrogenase (SDH), and SIRT 3 in aged liver tissue (Li et al., 2019b). Li et al. demonstrated that 6-gingerol (0.2 mg/kg) administered for 7 weeks to 22-month-old Sprague-Dawley rats attenuated age-associated elevations in triglycerides, glucose, and insulin. Additionally, it improved mitochondrial function, promoted a fast-to-slow muscle fiber transition, and enhanced oxidative metabolism in the red gastrocnemius muscle (Liu et al., 2019).

Beyond glucose regulation, gingerol shows protective effects against diabetes-related complications such as cardiopathy, kidney failure, and vision impairment (Alharbi et al., 2022). In STZ-induced diabetic models, 6-gingerol (10 mg/kg b. w.) reduced kidney fibrosis and pathological changes by lowering TNF-α expression (Almatroodi et al., 2021). Salah et al. reported that 6-gingerol improved vasoconstriction and nitric oxide generation in isolated aortae from STZ-induced diabetic mice (Ghareib et al., 2015). Moreover, 6-gingerol suppressed advanced glycation end product-induced ROS production in rat pancreatic β-cell line, RIN-5F and enhanced glucose uptake in L6 muscle cells via AMPK activation (Son et al., 2015).

Collectively, these findings indicate that 6-gingerol exerts multifaceted protective effects against diabetes and its complications through diverse mechanisms. Nonetheless, further large-scale, well-designed clinical trials are needed to fully establish its therapeutic efficacy and safety in diabetic populations.

### Mulberry

Mulberry leaves have traditionally been used for raising silkworms and as herbal remedies and beverages. Evidence from *in vitro*, *in vivo* and some clinical studies supports their potential health benefits. Mulberry leaves contain polyphenolic compounds, flavonoids, anthocyanins, and carotenoids (Chen et al., 2023), and have potent anti-inflammatory, anti-oxidant, anti-cancer, and anti-obesity activities (Ma et al., 2022a).

Bahram et al. reported that supplementation with mulberry leaf extract (MLE, 1,000 mg/day for 2 months) in older adult men with T2DM (aged 65–70 years) significantly reduced the expression of salusin-β and IL-6, both markers of inflammation. The effects were more pronounced in the group that combined exercise training with extract supplementation (Bahram et al., 2023).

In a randomized controlled study, a combination of mulberry leaf and white kidney bean extract (1.5 g per meal, daily for 4 weeks) significantly reduced postprandial glucose, insulin, and C-peptide levels in prediabetic participants aged 45–65 years (Liu et al., 2020). A randomized crossover trial involving 30 T2DM patients showed that MLE (250 mg containing 12.5 mg 1-deoxynojirimycin (DNJ)) reduced the 3-h glucose response (incremental area under the curve) by 15% when combined with fiber and chromium (Mohamed et al., 2023). Another study reported that evening intake of MLE (6 mg DNJ) improved 2-h postprandial glucose by 18% compared to morning intake in healthy young adults (Takahashi et al., 2023).

Numerous studies have investigated the hypoglycemic potential of mulberry leaf and fruit extracts using various *in vivo* and *in vitro* models (Chen et al., 2023). In diabetic db/db mice, dietary supplementation with mulberry fruit extract (MFE) for 12 weeks significantly reduced HbA1c and improved insulin resistance, as evidenced by enhanced HOMA-IR and upregulation of hepatic insulin signaling markers, including insulin receptor substrate (IRS)-1, p-AKT, and p-AMPK (Choi et al., 2016). MFE also exerted cytoprotective effects in pancreatic β-cells by inhibiting H_2_O_2_-induced ROS production and lipid peroxidation, thereby enhancing cell viability (Lee et al., 2014).

Mulberry leaf extracts, particularly those rich in alkaloids like 1-deoxynojirimycin, have shown strong inhibitory activity against intestinal α-glucosidase (Asano et al., 1994), resulting in reduced blood glucose levels in STZ-induced diabetic mice (Hu et al., 2019). Prolonged administration of aqueous mulberry leaf extract (1,200 mg/kg/day for 6 weeks) led to significant improvements in glycemic control, lipid profiles (reduced LDL-cholesterol and aspartate aminotransferase (AST)/alanine aminotransferase (ALT) ratio), and oxidative stress markers. Histological analysis also showed regeneration of pancreatic and hepatic tissue (Luo et al., 2023). Furthermore, dietary mulberry leaf powder preserved pancreatic β-cell mass and decreased markers of endoplasmic reticulum stress in db/db mice (Suthamwong et al., 2020). Antioxidant assays have demonstrated that mulberry leaf extracts possess potent free radical scavenging activity, surpassing even that of ascorbic acid (Ranjan et al., 2017).

*In vitro* studies further support these findings. Mulberry leaf flavonoids enhanced glucose uptake and glycogen synthesis in insulin-resistant human liver cancer cell line, HepG2 cells, restored mitochondrial membrane potential, and upregulated antioxidant enzymes such as SOD and catalase (Lv et al., 2022). In 3T3-L1 adipocytes, mulberry leaf flavonoids promoted GLUT4 translocation and exerted anti-apoptotic effects via modulation of the advanced glycosylation end-product specific receptor (AGE)-receptor for advanced glycation endproducts (RAGE) signaling pathway (Li et al., 2022).

Collectively, these findings indicate that mulberry leaf extracts exert more pronounced antidiabetic effects than fruit extracts, particularly at higher doses and with long-term use.

In diabetic nephropathy, mulberry leaf and fruit extracts reduce hyperglycemia, ameliorate renal dysfunction by lowering serum urea and creatinine levels, and suppress oxidative stress and inflammation in kidney tissues. These effects help mitigate glomerular sclerosis and renal fibrosis by downregulating proinflammatory cytokines such as TNF-α (Abouzed et al., 2020). Furthermore, mulberry extracts exhibit protective effects against diabetic retinopathy by preventing glucose-induced oxidative damage in retinal cells, primarily through antioxidant mechanisms involving anthocyanins like cyanidin-3-glucoside (Sukboon et al., 2025). However, most mechanistic studies have utilized cells derived from young animals, underscoring the need for future research employing aged cell models to better assess the therapeutic potential of mulberry in aging-associated diabetes.

### Myricitrin

Myricitrin, a glycosyloxyflavone found in *Myrica esculenta* bark, has demonstrated potential in mitigating age-related diabetes and its complications through various mechanisms. However, clinical trials evaluating the preventive and therapeutic effects of myricitrin in elderly diabetic patients or older adult populations have not yet been identified.

Aged mice induced by D-galactose (500 mg/kg/day) and co-treated with myricitrin (20 mg/kg/day) for 28 days showed improved insulin sensitivity and reduced insulin resistance. Enhanced β-cell function and increased islet diameter were observed in myricitrin-treated mice, with effects comparable to co-treatment with vitamin D (100 mg/kg/day). Moreover, myricitrin improved ALT and glutamic-oxaloacetic transaminase 1 (SGOT) levels and ameliorated histopathological liver changes induced by D-galactose, suggesting that myricitrin exerts both antidiabetic and hepatoprotective effects in aging-induced diabetic models.

Zhang B et al. demonstrated that oral administration of myricitrin (300 mg/kg/day for 8 weeks) improved diastolic dysfunction and attenuated histological abnormalities by decreasing the expression of cardiomyopathy-related enzymes, inflammatory cytokines, and apoptotic proteins. These cardioprotective effects were associated with activation of the Nrf2 pathway and inhibition of the nuclear factor kappa-light-chain-enhancer of activated B cells (NF-κB) pathway (Zhang et al., 2017).

Myricitrin was also found to attenuate diabetes-triggered renal inflammation by suppressing NF-κB activation. It inhibited hyperglycemia-induced apoptosis and fibrosis in renal cells, as evidenced by changes in the expression of apoptotic and fibrotic factors.

Additionally, myricitrin improves impaired nerve functions in diabetic peripheral neuropathy by reducing oxidative stress, advanced glycation end-products (AGEs), and enhancing antioxidant enzyme activities and nerve blood flow (Ma et al., 2022b). In diabetic retinopathy models, myricitrin protects retinal cells from high glucose-induced apoptosis and oxidative damage, partly through inhibition of signaling pathways like Sp1 and ERK (Pyun et al., 2017).

In future studies, clinical trials evaluating the effects and safety of myricitrin in humans are necessary. In particular, preliminary trials should assess its safety, pharmacokinetic properties, and appropriate dosage in human. Through this, the potential of myricitrin as a therapeutic agent can be verified.

### Quercetin

Quercetin (3, 3′, 4′, 5, 7-pentahydroxyflavone) is a natural compound with antioxidant potential (Forni et al., 2019). It is one of the most effective antioxidants in the flavonoid family and is found in kale, onions, berries, apples, red grapes, broccoli, cherries, and tea (Boots et al., 2008). Quercetin has also been extracted from many herbs such as *Euonymus alatus*, *Nelumbo nucifera*, *Ginkgo biloba*, *Morus alba,* and *Phoenix dactylifera* (El-Far et al., 2016; El-Far et al., 2019).

Oral administration of quercetin (250 mg/kg) improved antioxidant status in T2DM (Yi et al., 2021). In a clinical study, oral administration of quercetin (250 mg/day for 8 weeks) in patients with T2DM (aged 30–60 years) reduced maltose-induced postprandial hyperglycemia, though no significant effect was observed on glucose-induced postprandial hyperglycemia. However, Yao et al. reported that daily intake of quercetin (20.9 ± 2.32 mg/day) was associated with a reduced prevalence of T2DM in the Chinese population (Yao et al., 2019). The variation in clinical trial outcomes could be attributed to differences in dosage or the duration of the intervention, both of which may critically influence the clinical efficacy of quercetin.

In a senolytic combination therapy study (Dasatinib 100 mg + Quercetin 1,000 mg), a phase 1 trial in diabetic kidney disease patients (n = 9) reported reduced senescent cells in adipose tissue, evidenced by decreased p16INK4A-positive cells and senescence-associated secretory phenotype factors (Hickson et al., 2019). A phase 1 trial in Alzheimer’s disease was also completed, though further analysis is required to evaluate its relevance to diabetes.

In a D-galactose-induced aging model, rats supplemented orally with quercetin (25, 50, and 100 mg/kg for 42 days) showed dose-dependent reductions in aging, apoptotic, and inflammatory markers in the pancreas and kidneys (El-Far et al., 2020). Jeong et al. reported that low (0.04%) and high (0.08%) doses of quercetin administered for 6 weeks in db/db mice reduced blood glucose levels by 12% and 18%, respectively, with increased plasma adiponectin observed in the high-dose group compared to controls (Jeong et al., 2012).

In both pre-adipocytes and differentiated 3T3-L1 adipocytes, quercetin (20 μM) reduced the number of senescence associated (SA)-β-galactosidase-positive cells and suppressed inflammatory cytokines (IL-6 and TNF-α) and miR-155-5p expression induced by H_2_O_2_ (Zoico et al., 2021). Furthermore, treatment with 30 μM quercetin restored cell viability reduced by H_2_O_2_ in rat insulinoma cells, INS-1 pancreatic β-cells, an effect associated with increased glutathione peroxidase (GPX) and SOD activity (Kwon MJ et al., 2007).

Quercetin has demonstrated multifaceted protective effects against diabetic complications, particularly in mitigating vascular and renal damage. It exerts potent antioxidant and anti-inflammatory actions, reducing oxidative stress and inflammatory cytokines such as TNF-α and CRP, thereby improving vascular function and preventing exaggerated vasoconstriction in STZ or fructose induced rats (Mahmoud et al., 2013). In diabetic nephropathy, quercetin protects kidney function by inhibiting ferroptosis—a form of iron-dependent cell death—through activation of the Nrf2 signaling pathway, which enhances antioxidant defenses, decreases lipid peroxidation, and ameliorates renal tissue injury (Zhang et al., 2024). Additionally, quercetin improves nerve function and retinal health via modulation of molecular pathways like AMPK and NF-κB, further addressing diabetes-induced neuropathy and retinopathy (Saikia et al., 2024).

Taken together, quercetin demonstrates antioxidant and anti-inflammatory properties in preclinical models, suggesting its therapeutic potential for aging-driven diabetes and its complications, although further clinical studies are warranted to confirm its efficacy and optimal dosing.

### Resveratrol

Resveratrol is a polyphenolic compound primarily found in grape skin, peanuts, and berries. It has been reported to help improve age-related diabetes through its antioxidant, anti-inflammatory, and insulin-sensitizing effects. Supplementation with 150 mg/day of resveratrol for 1 month in obese men improved insulin sensitivity, mitochondrial function, and lipid metabolism (Timmers et al., 2011). In muscle tissue, resveratrol activated AMPK, subsequently enhancing glucose uptake and utilization in both liver and muscle, an effect mediated by SIRT 1 activation (Timmers et al., 2011).

Brasnyó et al. (2011) conducted a clinical trial in older adults with T2DM, administering resveratrol (5 mg in gelatin capsules, twice daily) over a 4-week period. The results demonstrated a significant reduction in fasting blood glucose and HbA1c levels, indicating improved glycemic control (Brasnyo et al., 2011). Clinical trials have demonstrated that resveratrol is well tolerated in older adult patients with T2DM. For instance, a study reported that a daily dose of 500 mg was well tolerated without significant adverse effects. However, some studies have noted that higher doses, particularly above 1,000 mg per day, may increase the risk of gastrointestinal symptoms such as diarrhea and abdominal discomfort (Smoliga et al., 2013).

Animal studies strongly support the efficacy of resveratrol in preventing age-related diabetes and suppressing its complications. In STZ-induced diabetic rats, intraperitoneal injection of 55 mg/kg resveratrol for 30 days reduced cholesterol and triglyceride levels and increased catalase and SOD levels (Schmatz et al., 2012). Moreover, treatment with 40 mg/kg resveratrol for 24 weeks reduced vascular inflammation markers, including NF-κB and IL-1β in blood (Zheng et al., 2013). Administration of 20 mg/kg for 12 weeks in *db/db* mice protected pancreatic islets from oxidative stress (Lee et al., 2012).

In HepG2 cells, high glucose levels induced the expression of inflammatory cytokines (NF-κB, TNF-α, IL-6, IL-1β and COX2), while co-treatment with resveratrol reduced these expression levels and improved glucose metabolism (Tshivhase et al., 2024). Resveratrol-treated cells showed activation of SIRT 1, inhibition of mammalian target of rapamycin complex (mTORC1), enhancement of antioxidant activity, inhibition of NF-κB, and improved mitochondrial function (Nanjan and Betz, 2014).

Resveratrol has shown protective effects against multiple diabetic complications, including nephropathy, neuropathy, and retinopathy, primarily via its antioxidant, anti-inflammatory, and metabolic regulatory actions. In diabetic nephropathy, resveratrol improves renal function by reducing oxidative stress, inhibiting inflammation, promoting autophagy, and activating AMPK pathways, which mitigate kidney damage induced by hyperglycemia. It also lowers levels of pro-inflammatory cytokines and advanced glycation end-products (AGEs), preventing renal fibrosis and dysfunction (Gowd et al., 2020). Additionally, resveratrol improves glucose metabolism, insulin sensitivity, and mitochondrial function, which further supports its role in preventing diabetic complications (Zhang et al., 2021b).

The results of cell-based studies demonstrate that resveratrol can alleviate diabetes-related cellular damage by suppressing inflammation, improving insulin signaling, and reducing oxidative stress. Based on these findings, various studies have confirmed that resveratrol is a promising natural compound for the prevention and treatment of age-related diabetes and its complications. Despite promising findings from animal and *in vitro* studies, clinical evidence remains limited, indicating that further human trials are needed to establish therapeutic protocols.

### Saffron (Crocus sativus L.)

Saffron is a legendary aromatic medicinal plant whose stigmas yield saffron, also called “red gold.” Saffron is rich in carotenoids and terpenes, with its major components being crocins and crocetin (carotenoids derived from zeaxanthin), as well as picrocrocin and safranal, which contribute to its taste and aroma, respectively.

In a randomized double-blind clinical trial, saffron supplementation (15 mg/day for 3 months) significantly decreased fasting plasma glucose, HbA1c, total cholesterol, and LDL cholesterol in T2DM patients (mean age: 53.5 years) compared to the non-treated group (Moravej Aleali et al., 2019). However, in another randomized controlled trial, saffron supplementation (100 mg/day, mean age: 54.1 years) for 12 weeks showed no significant difference in serum insulin, fasting blood glucose, HbA1c, or lipid profile (Ebrahimi et al., 2019). This discrepancy may be attributed to differences in dosage, study duration, and participant characteristics (such as the degree of insulin resistance).

Saffron has powerful antioxidant and anti-inflammatory properties in both *in vitro* and *in vivo* studies. In HFD-fed Sprague-Dawley rat models, saffron (40 and 80 mg/kg) improved insulin levels and lipid profile; its effect is associated with a decrease in oxidative stress and normalization of adiponectin levels (Mohaqiq et al., 2020). In fructose-fed rats, which developed insulin resistance, hyperinsulinemia, and dyslipidemia, treatment with crocetin significantly increased adiponectin expression while reducing TNF-α and leptin levels (Xi et al., 2007).

Saffron extract and crocins have shown promise in managing diabetic retinopathy. Saffron extract shows antioxidant effects by reducing lipid peroxidation in retinal tissue (Skourtis et al., 2020), while crocins exert antioxidant and anti-inflammatory properties via activation of the PI3K/AKT signaling pathway (Yang et al., 2017). Treatment with safranal reduced p38-AKT phosphorylation and the expression of cell adhesion molecules including E-cadherin, Snail, Twist, and fibronectin in high glucose-treated human retinal microvascular endothelial cells (Xiao et al., 2022). Additionally, saffron extract promoted autophagy in retinal ganglion cells and inhibited amyloid-β aggregation (Fernandez-Albarral et al., 2020). Recently, a combination of saffron, elderberry and *Melilotus officinalis* protected retinal pigment epithelial (ARPE-19) cells from oxidative stress by reducing caspase-1 activation and IL-1β secretion in H_2_O_2_-treated ARPE-19 cells (Puddu et al., 2025).

Saffron exhibits promising protective effects against diabetic complications by improving glycemic control, reducing oxidative stress, and modulating inflammation. Saffron shows neuroprotective effects against diabetic neuropathy by reducing glucose-induced oxidative damage and preserving nerve cell viability (Samarghandian et al., 2014). In diabetic retinopathy models, saffron extract enhances antioxidant defenses, protecting retinal cells from oxidative injury (Skourtis et al., 2020).

Together, these findings highlight saffron as a potential adjunct therapeutic agent for managing diabetes and its vascular, renal, neural, and retinal complications, though further well-controlled long-term clinical trials are needed to establish efficacy and dosing protocols.

## Discussion

Despite promising findings, several research gaps remain in our understanding of natural bioactive compounds for diabetes management in elderly populations. Notably, there is a lack of large-scale, randomized controlled trials specifically targeting frail elderly patients, and limited data on long-term safety and optimal dosing in this group. Filling these gaps through rigorous clinical and mechanistic studies will be essential to translate current evidence into clinical practice. Such efforts will provide a stronger evidence base to guide treatment guidelines, enabling the incorporation of these compounds as adjunctive options tailored to the elderly, thereby improving therapeutic efficacy and minimizing adverse effects in this vulnerable population.

Ethical considerations play a critical role in the management and application of natural products, particularly in elderly populations. It is imperative to ensure that these products meet strict standards of quality, safety, and efficacy, supported by robust scientific evidence. Patients must be provided with comprehensive information regarding potential benefits and risks to facilitate informed decision-making and uphold patient autonomy. While respecting cultural and traditional medicinal practices, regulatory frameworks must be in place to safeguard public health and guarantee product transparency. Furthermore, effective communication and patient education are essential to optimize therapeutic outcomes and minimize adverse effects. Adherence to these ethical principles is fundamental to the responsible and safe integration of natural products into clinical care.

This study has several limitations that warrant further consideration. The inherent variability and standardization challenges of natural products may affect reproducibility. Additionally, the studies and papers that were cited might be biased, which could limit the range of information included in the review. Acknowledging these limitations provides a balanced context for interpreting the findings and highlights the need for more rigorous future research.

Finally, while the primary focus of this study is elderly-onset diabetes, the implications of natural bioactive compounds are not confined to older populations. Frail elderly individuals often have reduced physiological reserve, greater susceptibility to hypoglycemia, and lower tolerance for polypharmacy or intensive pharmacological therapy, making relatively natural, plant-derived agents such as traditional herbal or botanical remedies particularly suitable as gentler and better-tolerated adjuncts. However, evidence also suggests benefits for younger at-risk populations. For example, mulberry leaf extract has been shown to reduce postprandial glucose and insulin levels by approximately 40% in normoglycemic young adults (Thondre et al., 2021), while a meta-analysis of randomized controlled trials—including participants with metabolic syndrome, often younger adults—demonstrated that saffron intake modestly reduced fasting blood glucose and HbA1c (Zhang et al., 2025). Collectively, these findings indicate that natural bioactive compounds are generally well tolerated and hold translational potential across both younger and older populations, though their clinical utility may differ by age group and physiological context.

## Conclusion

Elderly-onset T2DM presents distinct clinical and pathophysiological characteristics compared to diabetes diagnosed at younger ages, including a milder hyperglycemic profile, relatively preserved β-cell function, and unique risks for both microvascular and macrovascular complications. Management in this population is complicated by age-related physiological changes, multiple comorbidities, and an increased vulnerability to adverse drug effects, necessitating individualized and cautious therapeutic strategies.

**FIGURE 2:**
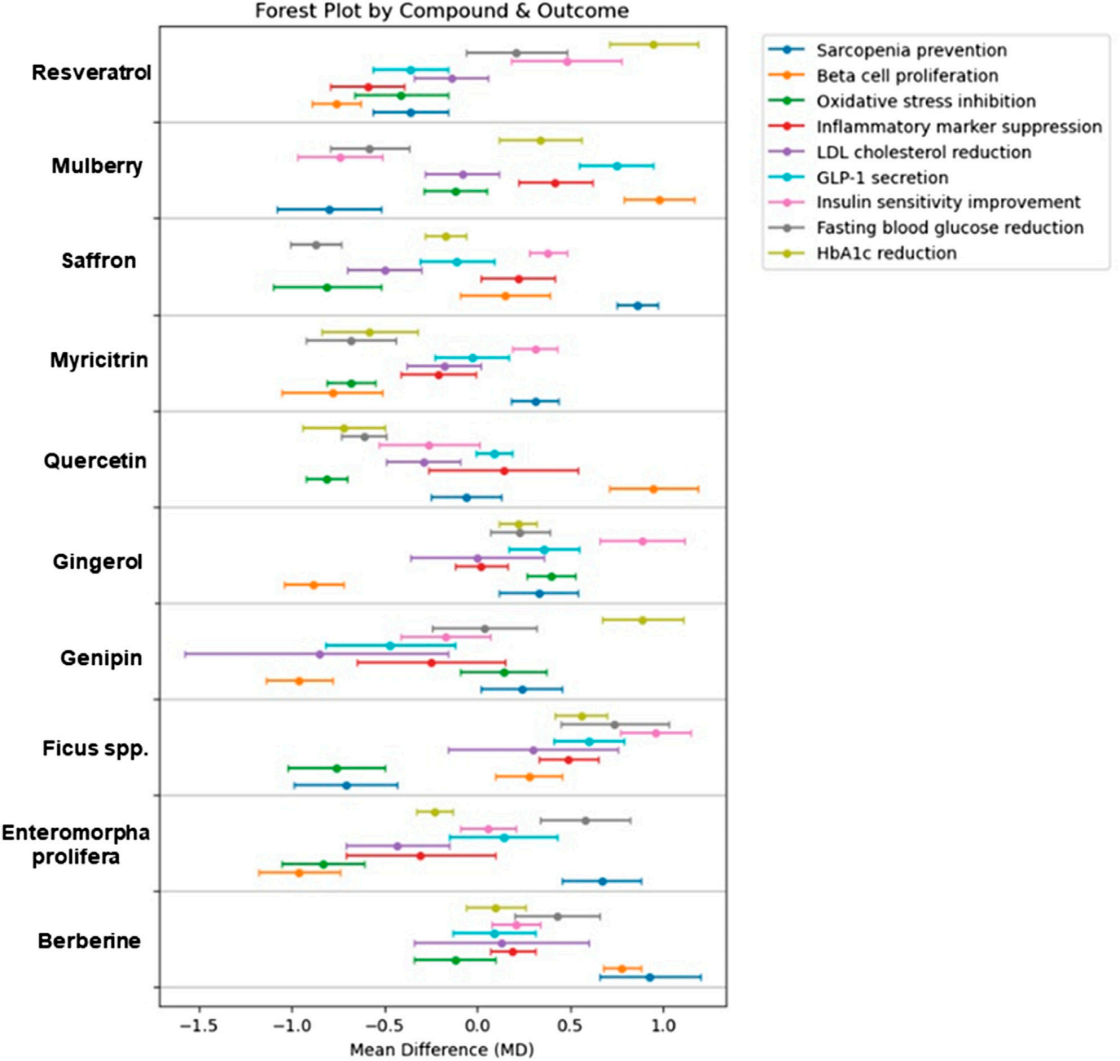
Forest plot of natural products targeting elderly diabetes Forest plot showing mean differences (MD) ± 95% confidence intervals for nine outcome measures HbA1c reduction, fasting blood glucose (FBG) reduction, insulin sensitivity improvement, GLP-1 secretion enhancement, LDL cholesterol reduction, inflammatory marker suppression (TNF-α), oxidative stress inhibition, β-cell protection, and sarcopenia prevention across ten natural products. Data were extracted from clinical and preclinical studies included in the review, with effect sizes calculated as the mean difference between treatment and control groups. Where multiple studies were available for the same natural product–outcome combination, results were normalized to baseline values and pooled using weighted averages. Negative MD values indicate beneficial reductions for outcomes where lower values are favorable (e.g., HbA1c, LDL cholesterol), whereas positive MD values indicate increases, which may be beneficial (e.g., insulin sensitivity, GLP-1 secretion) or undesirable depending on the parameter. Marker shapes distinguish outcome measures; horizontal bars indicate 95% CIs. The vertical line at zero represents no effect; CIs not crossing zero denote statistical significance.

**FIGURE 3:**
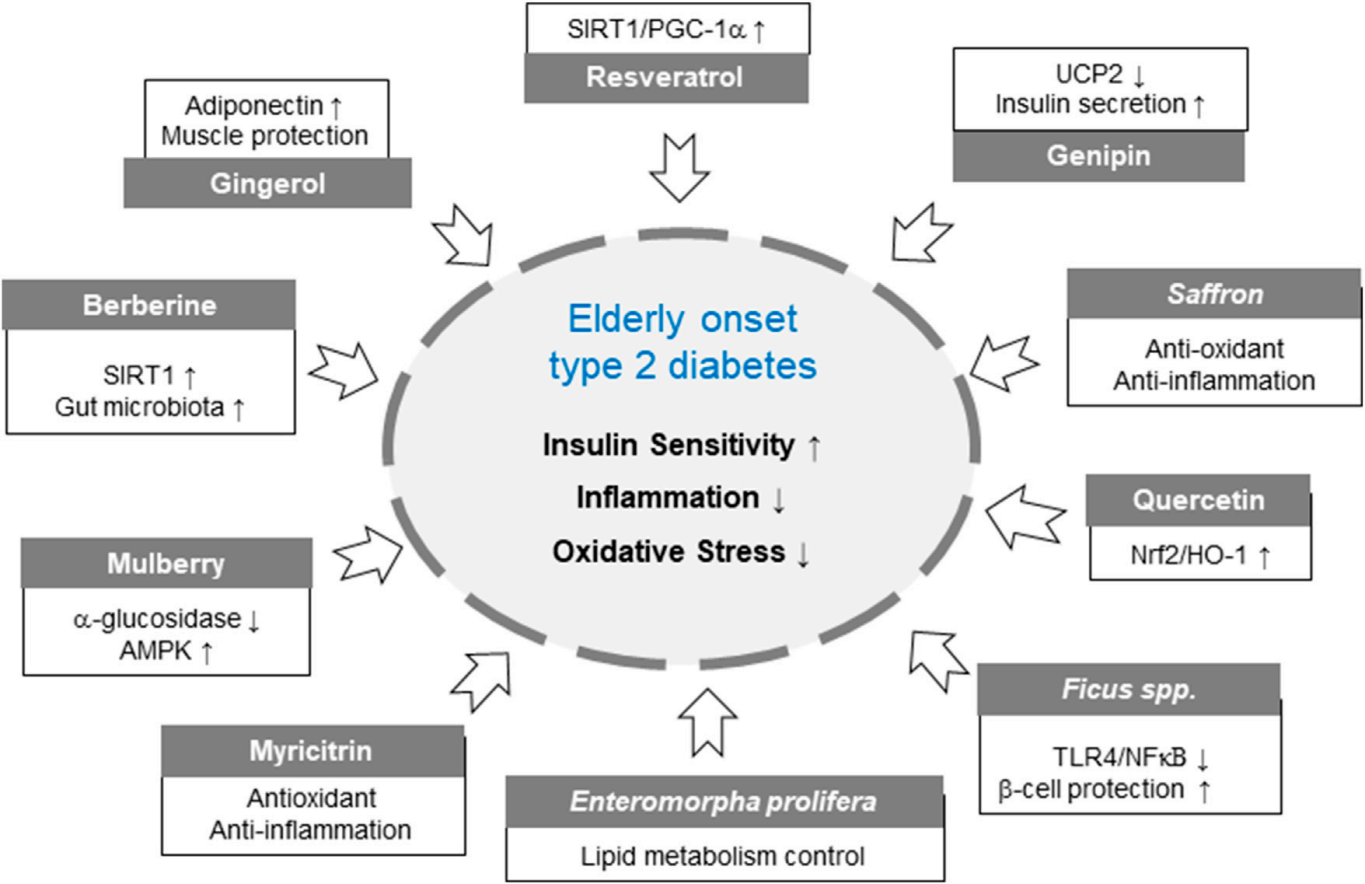
Common and specific Mechanisms of natural products targeting elderly diabetes Square refers to a specific mechanism, and circle refers to a general mechanism.

Emerging evidence suggests that bioactive compounds derived from natural sources—such as *E. prolifera* oligosaccharide, *Ficus* species, genipin, gingerol, mulberry, myricitrin, quercetin, resveratrol, and saffron—offer promising benefits for glycemic control and the mitigation of diabetes-related complications through diverse mechanisms, including antioxidant, anti-inflammatory, and insulin-sensitizing effects (Table 3). In summary, various natural compounds—including berberine, resveratrol, gingerol, mulberry, saffron, ficus, enteromorpha, myricitrin, genipin, and quercetin—demonstrated overall beneficial effects on metabolic health markers, such as HbA1c and fasting blood glucose reduction, improved insulin sensitivity, as well as decreased inflammation and oxidative stress (Figure 3). Most interventions consistently showed negative effect sizes, reflecting improvements across multiple outcomes, with some compounds exhibiting more pronounced efficacy for specific endpoints. Collectively, these findings highlight the potential of diverse natural products as supportive agents for the prevention and management of diabetes and related metabolic disorders (Figure 2). While conventional antidiabetic drugs remain indispensable, their limitations in elderly patients highlight the importance of developing safer, multi-targeted complementary options. To date, however, most supporting data derive from preclinical studies or small-scale clinical trials, highlighting the urgent need for large, well-designed human studies in older adult populations.

Importantly, this study emphasizes the translational potential of natural bioactive compounds to improve patient outcomes. Their favorable safety profiles and mechanistic diversity suggest meaningful opportunities to enhance glycemic control, mitigate diabetes-related complications, and improve quality of life in older adults when integrated thoughtfully with existing therapeutic strategies.

Beyond clinical implications, these findings also underscore the broader interdisciplinary significance of this field. Advancing research at the intersection of nutritional biochemistry, pharmacology, and geriatric medicine will be essential for developing personalized, safe, and multi-targeted interventions. Such cross-disciplinary efforts can accelerate the translation of promising natural products into evidence-based practice, ultimately fostering more effective strategies for diabetes management in aging populations.
